# Effect of plasma surface treatment of three different CAD/CAM materials on the micro shear bond strength with resin cement (A comparative in vitro study)

**DOI:** 10.1016/j.heliyon.2023.e17790

**Published:** 2023-06-29

**Authors:** Shahad Jabbar Jassim, Manhal A. Majeed

**Affiliations:** Aesthetic and Restorative Dentistry Department, College of Dentistry, University of Baghdad, Baghdad 10011, Iraq

**Keywords:** PiezoBrush®PZ3, Ceramic CAD/CAM materials, Micro shear bond strength, Zirconia, Lithium disilicate, Cerasmart, Surface roughness

## Abstract

**Objectives:**

This study aimed to evaluate and compare the effect of plasma treatment versus conventional treatment on the micro shear bond strength (μSBS), surface roughness, and wettability of three different CAD/CAM materials.

**Materials and methods:**

Sixty cylindrical specimens (5 mm diameter × 3 mm height) were prepared from three different CAD/CAM materials: Group A: Zirconia, Group B: Lithium disilicate, and Group C: Resin nano-ceramic. Each group was subdivided into two subgroups according to surface treatment used: Subgroup I: Conventional treatment, zirconia was sandblasted with Al_2_O_3_, while lithium disilicate and resin nano-ceramic were etched with hydrofluoric acid. Subgroup II: Plasma treatment, the surface of each material was treated with a plasma device (PiezoBrush® PZ3 Handheld Device, Relyon Plasma, Regensburg, Germany). G-Multi PRIMER was applied, then self-adhesive cement (G-CEM ONE) was applied using a split mold (1 mm diameter × 3 mm height), and μSBS was tested in a universal testing machine. The surface roughness was measured using a profilometer. Nine additional specimens of each material for wettability test using an optical tensiometer.

**Statistical analysis:**

The data were analyzed using ANOVA and Bonferroni test at a level of significance of 0.05.

**Results:**

The highest mean of μSBS was recorded by AII (27.3 MPa), while the lowest was recorded by AI (17.9 MPa). One-way ANOVA test revealed a significant difference among groups. Bonferroni test showed each two subgroups significant difference except subgroups AI, CI and BII, CII, where there was a non-significant difference. For all CAD/CAM materials, conventional treatment increased the surface roughness compared to plasma treatment, while the contact angle decreased after plasma treatment.

**Conclusion:**

Plasma treatment increased the μSBS of resin cement to zirconia significantly while not significantly affecting the μSBS of resin nano-ceramic. Conventional treatment of lithium disilicate provided significantly higher μSBS than plasma treatment.

## 1 Introduction

All ceramic CAD/CAM materials are manufactured, producing extremely homogeneous materials with superior mechanical characteristics under standardized and ideal conditions as compared to laboratory-processed materials. These materials can be generally categorized into three main ceramic groups depending on their formulation: polycrystalline, glass matrix, and resin matrix [1,2]. Yttria-stabilized tetragonal zirconia polycrystals (Y-TZP) are polycrystalline ceramics that lack a glass matrix [3]. Zirconia is a bioinert and biocompatible ceramic with high esthetics, low thermal conductivity, excellent mechanical strength, and chemical stability. Zirconia, on the other hand, has a low surface energy [4].

Lithium disilicate ceramic is one of the glass matrix ceramic types that can be used to fabricate monolithic restorations; it has superior esthetics and excellent physical characteristics [5,6]. Nevertheless, adhesive cementation is essential because lithium disilicate prosthetics are prone to fracture from chewing forces [7].

Another method for optimizing CAD/CAM materials is to create a resin-matrix. CAD/CAM blocks are particularly associated with novel microstructures containing dispersed fillers, known as resin nano-ceramics [8]. The goal of material development was to create a material that milled easily and could be repaired intra-orally more easily while also being more similar to dentin's elasticity modulus [2]. The chemical composition and microstructure of the CAD/CAM material used for constructing the monolithic crowns had an important influence on their fracture strength [9] and also influenced by various surface treatment techniques. It is essential to choose the pretreatment type, concentration, exposure duration, and cleaning technique [10].

It is well-known that the surface treatment of ceramic restoratives before cementation effectively improves the prognosis [11,12]. Hydrofluoric acid (HF) positively enhances the surface morphology of the lithium disilicate and can also be used for resin nano-ceramic materials [13,14]. HF etchant acts by dissolving the glass phase of the ceramic matrix and exposing the crystalline structure [15]. However, excessive HF exposure or incorrect application can result in microcracks in the material. Resulting in a decrease in flexural strength. Great caution is required when using hydrofluoric acid due to its higher corrosive power and toxicity [16]. The surface treatment usually used for zirconia is airborne particle 50 μm Al_2_O_3_. However, this may damage the surface of zirconia, leading to microcracks that might limit the prognosis [17].

On the other hand, zirconia still faces the problem of achieving durable adhesion to resin cement. Because of its chemical stability and hydrophobic properties with different substrates, zirconia bonding is a constant challenge [18].

In recent years, the plasma surface treatment of ceramic, considered a novel use in dentistry, has attracted much attention. The fourth state of matter, plasma, comprises atoms, molecules, and highly excited radicals. A thermal and non-thermal (cold) type with gas temperatures close to room temperature exists at atmospheric pressure [19]. Atmospheric plasma is used for surface activation, affecting the topmost atomic layers of the surface. An untreated surface has hardly any polar groups that could interact with the liquid, while plasma creates such polar end groups on the molecules of the surface. With the Piezobrush® PZ3 (Relyon Plasma, Regensburg, Germany), which is a handheld non-thermal air atmospheric pressure plasma device, this can be done almost without any thermal effect on the surface. After treatment, the surface still looks the same, but is now equipped with polar anchor groups that can form strong bonds with the adhesive. The plasma that produces carboxyl groups improves the wettability of the surface, making it hydrophilic [20,21,22].

Due to the aforementioned shortcoming of conventional surface treatment methods, this study was conducted to evaluate and compare the micro shear bond strength (μSBS) of resin cement to three different CAD/CAM materials after plasma surface treatment versus conventional surface treatment. The null hypotheses:

I. Plasma treatment will not increase the micro shear bond strength of resin cement to the different all ceramic CAD/CAM materials tested.
II. Plasma treatment will not increase the surface roughness of the different all ceramic CAD/CAM materials tested.
III. Plasma treatment will not increase the wettability of the different all ceramic CAD/CAM materials tested.

## 2 Materials and method

### 2.1 Specimen preparation

Three different ceramic CAD/CAM materials were used in this study, comprising three main groups:

Group A: Zirconia (IPS e.max ZirCAD, A2 LT, Ivoclar Vivadent, Schaan/Liechtenstein).

Group B: Lithium disilicate (IPS e.max CAD, A2 LT, Ivoclar Vivadent, Schaan/Liechtenstein).

Group C: Resin nano-ceramic (Cerasmart, A2 LT, GC Corp., Tokyo, Japan).

Twenty cylindrical specimens (5 mm diameter × 3 mm height) were prepared from each material [23]. Zirconia specimens were prepared to an enlarged size (20% larger) to compensate for the sintering shrinkage that would occur later on. The cutting process was done using a diamond disc bur (Star dental mart, India) with 40 mm diameter and 1 mm thickness attached to a low speed hand piece rotated counter clockwise with a rotational speed of 20,000 RPM under water cooling. The dimensions of each specimen were then checked using a digital caliper. Zirconia specimens were then sintered at 1550 °C for 120 min (a total time of approximately 8 h) in a sintering furnace (ZETIN, China). Following the sintering procedure, each zirconia specimen's dimensions were rechecked.

Specimens made from IPS e.max CAD (Ivoclar Vivadent) were subjected to a crystallization firing cycle in a ceramic firing furnace (Programat EP 3010, Ivoclar Vivadent \ technical, Liechtenstein, Germany) for approximately 30 min at 840 °C according to the manufacturer's instructions to impart the glass ceramic samples with their final strength and esthetic properties. On the other hand, cerasmart specimens were not subjected to any heat treatment as the material is supplied in a fully crystallized state.

The specimens of all groups were then individually mounted in acrylic blocks using a custom-made cylindrical silicone mold with internal dimensions of (18 mm × 18 mm). The mounting procedure was used a dental surveyor (TECHNIC, U.S.A.).

The bonding surface of each specimen was then polished with 600, 800, 1000, and 1200 grit silicon carbide paper under water coolant to provide a standardized roughness that resembled that produced after milling [23]. The specimens were then cleaned in an ultrasonic cleaner containing distilled water for 5 min to remove the loose particles of the ceramic materials [18]. Accordingly, Table 1 gives a description of the experimental materials used.

**Table 1:** The experimental materials used in this study.

Material	Manufacturer	Composition
Zirconia	IPS e-max ZirCAD LT, Ivoclar Vivadent, Schaan, Liechtenstein	Zirconium oxide (ZrO_2_) 88.0–95.5%, Yttrium oxide (Y_2_O_3_) > 4.5% – ≤ 6.0%, Hafnium oxide (HfO_2_) ≤ 5.0%, Aluminium oxide (Al_2_O_3_) ≤ 1.0%, Other oxides ≤1.0%
Lithium disilicate	IPS e.max CAD, Ivoclar Vivadent, Schaan, Liechtenstein	Lithium disilicate reinforced glass-ceramic of the Li_2_O–K_2_O–P_2_O_5_–MgO-material system
Resin nano-ceramic	Cerasmart, GC Dental Products, Tokyo, Japan	Composite resin material (BisMEPP, UDMA, DMA) with 71 wt% silica and barium glass nanoparticles
Hydrofluoric acid	IPS ceramic etching gel Ivoclar Vivadent	≤5% hydrofluoric acid
Universal primer	G-Multi PRIMER, GC Corporation, Tokyo, Japan	Ethanol, MDP, MDTP, silane, methacrylate monomer
Self-adhesive resin cement	G-CEM One, GC Corporation, Tokyo, Japan	Paste B: SiO_2_, trimethoxysilane, UDMA, 2-hydroxy-1,3-dimethacryloxypropane, MDP, 6-tert-butyl-2,4-xylenol, 2,6-di-tert-butyl-p-cresol, EDTA disodium salt dehydrate, vanadyl acetylacetonate, TPO, ascorbic acid, camphorquinone, MgO

Abbreviation: Bis-MEPP: 2.2-bis (4-methacryloxypolyethoxyphenyl) propane; UDMA: urethane dimethacrylate; DMA: dimethacrylate; MDP: methacryloyloxydecyl dihydrogen phosphate; MDTP: methacryloyloxydecyl dihydrogen thiophosphate; Silane: γ-methacryloxypropyl trimethoxysilane. UDMA: Urethane dimethacrylate, EDTA: ethylenediaminetetraacetic acid, TPO: thermoplastic polyolefin.

### 2.2 Surface treatment

The specimens of each group were further subdivided into two subgroups of 10 specimens according to the types of surface treatment used: Conventional surface treatment (Subgroups AI, BI, and CI), Plasma surface treatment (Subgroups AII, BII, and CII). The sample size was determined based on previous studies [23,24,25].

**Fig. 1:**
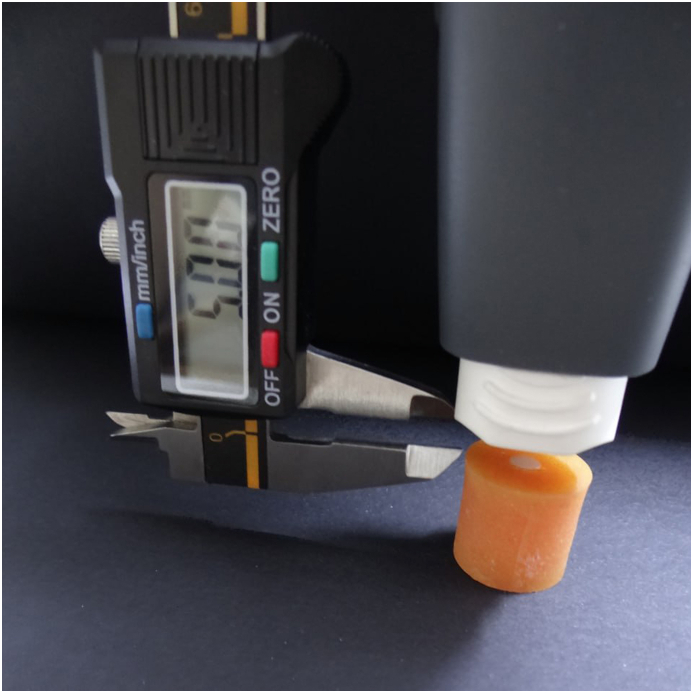
Non-thermal plasma device (PiezoBrush® PZ3 Handheld Device, Relyon Plasma, Regensburg, Germany) was fixed at a distance of 5 mm from specimen.

For conventional surface treatment, zirconia specimens were sandblasted with 50 μm Al_2_O_3_ at a distance of 10 mm for 10 s under 1 bar pressure, perpendicular to the zirconia surface. Specimens from IPS e.max CAD were etched with <5% hydrofluoric acid gel (IPS Ceramic Etching Gel, Ivoclar Vivadent, Schaan/Liechtenstein) for 20 s. The gel was then thoroughly rinsed with water for 15 s [26]. Cerasmart specimens were etched with <5% hydrofluoric acid gel for 60 s, the gel was then rinsed off with water for 1min according to the manufacturer's instructions. All specimens were then cleaned in an ultrasonic cleaner containing distilled water for 5 min, then air dried with an air spray until the surface was completely dry. For plasma surface treatment, the corresponding specimens of each subgroup were subjected to non-thermal plasma treatment for 80 s, 100% plasma power and 5 cm^2^/s treatment speed using PiezoBrush® PZ3 Handheld Device (Relyon Plasma, Regensburg, Germany) that generates an atmospheric pressure plasma jet. The nozzle of the plasma device was fixed 5 mm away from the specimen's surface, as shown in Fig. 1.

### 2.3 Cementation procedure

After completing the surface treatment of all specimens, an adhesive tape was applied to the surface of the specimen with a central hole of 1 mm. The tape was carefully applied to the ceramic disc in order to expose the surface treatment area through the perforation. G-Multi PRIMER (GC Corp., Tokyo, Japan) was then applied with a disposable brush to the exposed surface of each specimen, then air dried for 5 s according to the manufacturer's instructions. A split mold, specially-designed and fabricated for this study, with an internal diameter of 1 mm and a height of 3 mm [27] was then placed over the uncovered area, with its lumen coincided with the exposed area, then secured in position with the aid of a custom-made mounting jig.

Self-adhesive resin cement (G-CEM ONE, GC Corp., Tokyo, Japan) was then applied into the mold using a disposable automixing tip. Light cured for 10 s from above using eighteenth curing pen with light intensity of 1500 mW/cm^2^ (Eighteeth, China) following the manufacturing instructions. After that, the cured resin cement was left for 4 min to completely polymerize prior to the removal of the mold. The mounting jig was then released and the split mold and the tape were removed. A scalpel blade No. 11 was used to carefully remove excess resin cement from the area surrounding the resin cylinder. All specimens were examined under a digital microscope (Digital Microscope, Model X4, China) at a magnification of × 25 to verify no bonding defects, air bubbles present, interfacial gaps, or excess cement. All specimens were then stored in deionized distilled water in an incubator at 37ᵒC for 24 h before μSBS.

### 2.4 Micro shear bond strength test

Micro shear bond strength test was carried out using a universal testing machine (LARYEE, China) at a crosshead speed of 0.5 mm/min with a stainless steel knife-shaped blade [12]. The force was applied to the adhesive interface until it fractured, and the failure load was measured in Newton, which was then divided by the surface area (mm^2^) to calculate the shear bond strength in MPa (N/mm^2^).

μSBS= Force (Newton)/surface area (mm^2^)

Surface area = πr^2^

After μSBS test, all the fractured specimens were examined under a digital microscope to determine the type of failure as follows [28]:

Type I: adhesive failure; the fracture line was restricted within the ceramic or resin cement.
Type II: cohesive failure; the fracture line was restricted either within the ceramic material or the resin cement.
Type III: mixed failure; when the cohesive and adhesive failures occur simultaneously.

### 2.5 Surface roughness

The surface roughness of each specimen was initially measured before surface treatment, representing the baseline measurement, then measured again after the different surface treatments. A surface roughness tester (leeb 432A, Testcoat, USA) was used to measure surface roughness. The profilometer was used to measure the (Ra) for each specimen in (μm) according to ISO-DIN 4768 specification for surface roughness measurement [29], whereby a diamond stylus of 5 μm diameter and stylus angle 90° was traversed in a length of 1.25 mm and with cut-off length 0.25 mm and force <4 mN. Three measurements were taken in the center of each sample at crossing directions assisted by using a ruler, and then the average reading was taken [30].

### 2.6 Wettability test

Nine additional specimens were prepared from each material to be used for wettability testing. The sessile drop method was used to measure the wettability of each CAD/CAM material, whereby a drop of distilled water was dispensed at the surface of the material at room temperature, then the contact angle was measured after 10 s using an Optical Tensiometer (TL 1000, Theta Lite, OneAttension, Biolin Scientific, Lichfield, UK) [23,31]. For each material, three specimens were used (n = 3) and the contact angle was measured at three times:

(1) Before surface treatment (control), (2) After conventional surface treatment, and (3) After plasma surface treatment.

## 3 Results

### 3.1 Micro shear bond strength

Shapiro-Wilk test revealed that the data were normally distributed (P > 0.05). The descriptive statistics including the mean and standard deviation of the micro shear bond strength in (MPa) of the different subgroups are shown in Table .2.

**Table 2:** Mean ± standard deviations of μSBS in MPa.

Conventional surface treatment	Mean ± SD^a^	Plasma surface treatment	Mean ± SD
AI	17.9 ± 1.37^aA^	AII	27.3 ± 3.30^aB^
BI	21.75 ± 1.87^bA^	BII	19.5 ± 1.12^bB^
CI	18.36 ± 1.32^aA^	CII	19.2 ± 1.14^bA^

Note: Different superscript letters represent significant differences (lowercase letters for columns, uppercase letters for rows) (*P* < 0.05).

a SD, standard deviation.

The lowest mean value of the micro shear bond strength was recorded by group AΙ (17.9 MPa), while the highest mean value was recorded by group AII (27.3 MPa).

One-way ANOVA test was used for comparison of the micro shear bond strength among the different conventional and plasma surface treatment subgroups. This test revealed statistically significant differences (*P* < 0.05). Bonferroni test was then used for multiple comparisons between each two subgroups to show specifically where the significant differences occurred. This test showed statistically significant differences between subgroups AI and BI, BI and CI, AII and BII, and AII and CII (*P* < 0.05), while no significance different was found between subgroups AI and CI, and BII and CII (*P* > 0.05) (Table 2). Independed *t*-test for comparsion of micro shear bond strength showed statistically significant differences (*P* < 0.05), between AI and AII, BI and BII, and no significant different was found between CI and CII (*P* > 0.05) (Table 2).

The failure modes are presented in Table 3. That although specimens failed in cohesive, adhesive, and mixed failure modes, it was discovered that the adhesive was observed among most groups.

**Table 3:** Modes of failure of the different groups.

Groups	Subgroups	Adhesive	Cohesive	Mixed	Total (%)
A	AI	4 (40%)	2 (20%)	4 (40%)	10 (100%)
	AII	2 (20%)	6 (60%)	2 (20%)	10 (100%)
B	BI	2 (20%)	5 (50%)	3 (30%)	10 (100%)
	BII	6 (60%)	1 (10%)	3 (30%)	10 (100%)
C	CI	7 (70%)	1 (10%)	2 (20%)	10 (100%)
	CII	3 (30%)	2 (20%)	5 (50%)	10 (100%)

### 3.2 Surface roughness

The descriptive statistics including the mean and standard deviation of the surface roughness of the different subgroups are shown in Table 4. Before surface treatment, the lowest mean value of surface roughness was recorded by subgroup CII (0.251 μm), while the highest mean value was recorded by subgroup AII (0.344 μm). After surface treatment, it can be seen the lowest mean value of surface roughness was recorded by subgroup CIΙ (0.258 μm), while the highest mean value was recorded by group BI (0.8 μm). Bonferroni test showed statistically significant differences between subgroups before surface treatment AI and BI, AI and CI, AII and BII, and AII and CII (*P* < 0.05), while no significant difference was found between subgroups BI and CI, and BII and CII (*P* > 0.05). Bonferroni test showed statistically significant differences between subgroups after surface treatment AI and BI, BI and CI, AII and BII, and AII and CII (*P* < 0.05), while no significant different was found between subgroups AI and CI, and BII and CII (*P* > 0.05) (Table 4). Paired *t*-test for comparison of the surface roughness before and after conventional surface treatment showed a significant different (*P* < 0.05) for all groups, no significant difference before and after plasma surface treatment (*P* > 0.05) (Table 4).

**Table 4:** Mean ± standard deviations of surface roughness of the different subgroups in (μm) before and after surface treatment.

		Before surface treatment (μm)	After surface treatment (μm)
Conventional surface treatment	AI	0.323 ± .0271^aA^	0.677 ± .015^aB^
	BI	0.260 ± .019^bA^	0.8 ± .029^bB^
	CI	0.266 ± .0269^bA^	0.652 ± .024^aB^
Plasma surface treatment	AII	0.344 ± .0365^aA^	0.349 ± .0052^aA^
	BII	0.265 ± .0208^bA^	0.271 ± .019^bA^
	CII	0.251 ± .0185^bA^	0.258 ± .023^bA^

Note: Different superscript letters represent significant differences (lowercase letters for columns, uppercase letters for rows) (*P* < 0.05).

### 3.3 Wettability test

**Fig. 2:**
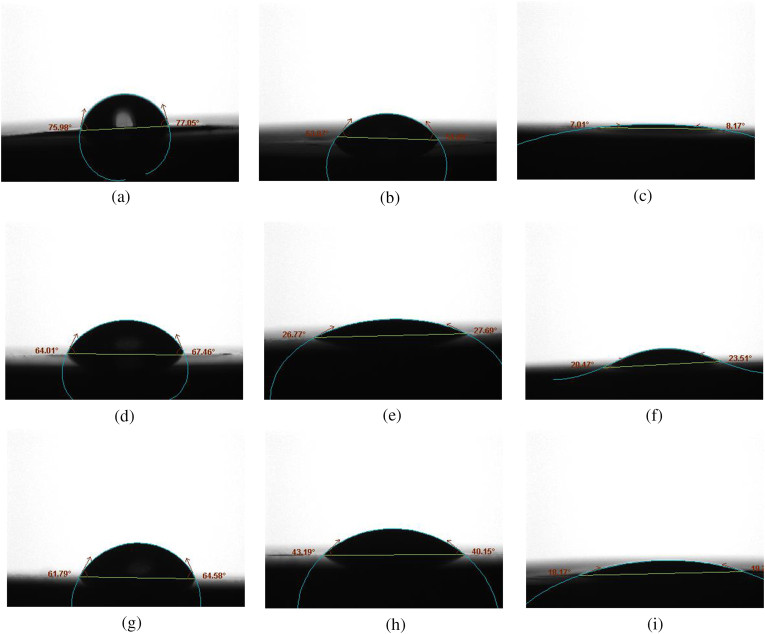
Representative photographs for the contact angle of the different materials before and after different surface treatment. (a): No surface treatment (zirconia). (b): After conventional surface treatment (AI). (c) After plasma surface treatment (AII). (d): No surface treatment (e.max CAD). (e): After conventional surface treatment (BI). (f) After plasma surface treatment (BII). (g): No surface treatment (cerasmart). (h): After conventional surface treatment (CI). (i) After plasma surface treatment (CII).

The averages of the contact angles of the different ceramic materials before and after surface treatment are shown in Table 5. Fig. 2(a–i) shows the contact angles of zirconia, e.max CAD and cerasmart, respectively, before and after the different surface treatments. The lowest average contact angle was seen in subgroup AII (7°), while the highest was seen in subgroup AI (76°).

**Table 5:** Average of contact angle of the different subgroups before and after surface treatment.

Groups	Subgroups	Contact angle average
A	Control	76^°^
	AI	54 ^°^
	AII	7 ^°^
B	Control	64.6 ^°^
	BI	29 ^°^
	BII	23.3 ^°^
C	Control	63 ^°^
	CI	41.3°
	CII	17.3°

## 4 Discussion

Each of the conventional surface treatment methods for CAD/CAM materials has its own shortcoming giving a rationale for searching for alternative methods that, apart from providing adequate bond shear, will not affect the microstructure of the treated materials.

In this study, three different materials belonging to the three main categories of all-ceramic CAD/CAM materials were used in this study. The selection of these materials was based on that these materials have different chemical compositions with different physical and mechanical properties.

The bonding strength between resin cement and the different CAD/CAM materials was evaluated using μSBS test as it gives higher percentage of adhesive specimen diameter, reducing the chance of complex stress generation, yielding more adhesive than mixed failures [32,33].

Two factors affect the bond efficiency of ceramic materials: micromechanical interlocking and chemical bonding [34]. The former is provided by the conventional surface treatment methods, including sandblasting and hydrofluoric acid etching, while chemical bonding is achieved via the use of a universal primer to promote bonding of the hydrophobic resin cement to the different CAD/CAM materials. G-Multi PRIMER was used because it is a universal primer containing three functional monomers (MDP, MDTP, and silane) for bonding to different substrates [14].

The results of this study revealed that plasma treatment of zirconia increased the μSBS significantly with no significant alteration of the surface roughness as compared to conventional surface treatment by sandblasting with aluminum oxide.

This could be attributed to that plasma surface treatment of zirconia surface with Piezobrush PZ3 increased the surface energy and produced a material surface that is clean, making it easier for heterostructures to connect effectively. By presenting oxygen to the surface of inert ceramic materials during plasma treatment, surface energy was enhanced, thereby facilitating the formation of reactive superoxide radicals (R-O-O-), thereby increasing the surface energy [35]. The increase in surface energy, in turn, increased the wettability of the surface as revealed by results of wettability test which showed that the contact angle of zirconia surface decreased from 76° to 7° after plasma treatment.

On other hand, the plasma can effectively remove contaminants and impurities from the surface [18]. The plasma's excited particles react with the surface layer of hydrocarbons and carbonate molecules, breaking the C–H and C–C bonds, thereby oxidizing them to form H_2_O and CO_2_, which are pumped away after desorbing from the surface [36]. However, It is important to remember that plasma treatment will neither change the crystalline structure nor cause phase transformation of zirconia as found by XRD and XPS [25,37]. In contrary, conventional surface treatment of zirconia by sandblasting with 50 μm Al_2_O_3_ causes subsurface damage and results in the formation of microcracks during the impaction of the powder particles that might compromise the mechanical strength at the surface layer [38]. This finding is in agreement with the results of Silva et al. [39], Ito et al. [37], Mahrous et al. [18], Silva et al. [40], and Ye et al. [41], who all found a significant increase in the SBS of resin cement to plasma-treated zirconia surface regardless of the type of plasma device, type of zirconia and cement used. However, this result disagrees with the findings of Mohd Azmi [33] and Ahn et al. [23], who found that plasma treatment did not increase the SBS of resin cement to zirconia. Such disagreement could be attributed to the differences in the type of plasma device used and the lack of a standard protocol regarding the duration, distance, and power used.

Regarding lithium disilicate, the results of this study showed that the conventional surface treatment of lithium disilicate with hydrofluoric acid provided significantly higher μSBS than plasma treatment. This could be attributed mainly to the increased surface roughness and wettability of the etched surface compared to the untreated and plasma-treated specimens. The gold standard for the treatment of silica-based ceramics has been considered to be hydrofluoric acid is used to etch lithium disilicate before being primed with a silane coupling agent [42]. Hydrofluoric acid reacts with the glass matrix forming hexafluorosilicates, which are then selectively removed, exposing the crystalline structure and providing a rough surface allowing for enhanced micromechanical retention [43].

Another possible reason for the superior bonding performance of resin cement to lithium disilicate could be attributed to the better chemical interaction between the hydrophobic resin cement and the e.max CAD surface. The roughly etched surface exhibits higher surface energy prior to combining with the ceramic primer [44]. This, in turn, will provide a covalent hydrogen bond, which will play a significant role in creating a sufficient resin bond to silica-based ceramics. This treatment method enables better micromechanical retention and/or more physical interactions with the generally hydrophobic luting resin [45].

This finding is in agreement with the results of Bitencourt [46], who found that the bond strength of resin cement to lithium disilicate after non-thermal plasma treatment was higher but comparable with that after conventional hydrofluoric acid treatment. However, this finding is in disagreement with Filho et al. [47] and Dos Santos et al. [48] who found higher surface energy and increased SBS after plasma treatment than in traditional hydrofluoric acid etching. However, conventional surface treatment of e.max surface with hydrofluoric acid still has hazardous effects and is highly corrosive material [49].

On the other hand, the results of this study showed that the μSBS of resin cement to lithium disilicate treated with hydrofluoric acid was significantly higher than that of resin cement to resin nano-ceramic. This could be attributed to the significantly higher surface roughness of etched lithium disilicate than that of etched resin nano-ceramic owing to the relatively higher silica content of lithium disilicate. This is in agreement with Şişmanoğlu et al. [50], who found that hydrofluoric acid treatment provided higher bond strength when the ceramic content in the material composition was increased.

Although statistically non-significant, the results of this study showed that plasma treatment of resin nanoceramic provided higher μSBS than hydrofluoric acid etching. This is in agreement with the results of Zhu et al. [51], who found that plasma treatment of Renci Upcera resin nanoceramic increased the SBS and wettability.

The results of this study elicited that the effect of plasma treatment is material dependent and needs further investigations. Therefore, the first null hypothesis was partially rejected as plasma treatment increased micro shear bond strength of resin cement to zirconia only, with a non-significant effect for other materials tested. In addition, the second hypothesis was accepted as plasma treatment did not significantly increase the surface roughness of all the materials tested, while the third hypothesis was rejected as plasma treatment significantly increased the surface roughness of the tested materials.

The more pronounced effect of plasma treatment on zirconia could be attributed to the higher initial surface roughness of this material compared to lithium disilicate and resin nano-ceramic as revealed by the results of this study. Thus, plasma treatment of zirconia restorations can be considered a promising surface treatment method as it provides higher μSBS than sandblasting while eliminating the risk of fissures and cracks that might occur with sandblasting [52].

Regarding lithium disilicate and resin nano-ceramic, plasma surface treatment can overcome the drawbacks of conventional surface treatment of hydrofluoric acid.

The present study's greater rate of adhesive failures may be related to the micro shear bond strength test required for small bonded cross-sectional areas (1 mm^2^ or less) [53,54]. When failures of the bond between the ceramic materials and resin cement are stronger than the resin cement itself, as shown by cohesive failures in the resin cement.

One of the limitations of this study is that the specimens had only one adhesive interface that is the luting resin-CAD/CAM material interface, and had not considered the other interface, which is the luting resin-tooth interface, which could affect the stress pattern during bond strength testing and may not represent the actual value clinically. Another limitation of this study was using of fixed parameters for the plasma device for all materials tested. However, in the absence of a standard protocol for the use of such handheld plasma device, further studies with variable settings for the plasma device are required to settle the optimum settings for each material.

## 5 Conclusion

Within the limitations of this *in vitro* study, the following conclusions could be drawn:

1 Plasma surface treatment significantly increased the μSBS of resin cement to zirconia, with a non-significant increase of μSBS to resin nano-ceramic, while conventional surface treatment of lithium disilicate with hydrofluoric acid provided significantly higher μSBS.
2 Plasma surface treatment did not significantly affect the surface roughness of the tested all-ceramic CAD/CAM materials.
3 Plasma surface treatment increased the wettability of the tested materials.

## Author contribution statement

Shahad Jabbar Jassim: Performed the experiments; Analyzed and interpreted the data; Contributed reagents, materials, analysis tools or data; Wrote the paper.

Manhal A. Majeed: Conceived and designed the experiments; Analyzed and interpreted the data; Contributed reagents, materials, analysis tools or data; Wrote the paper.

## Data availability statement

Data will be made available on request.

## Funding

The study is self-funded.

## Additional information

No additional information is available for this paper.

## Declaration of competing interest

The authors declare that they have no known competing financial interests or personal relationships that could have appeared to influence the work reported in this paper.
